# ROS signaling in innate immunity via oxidative protein modifications

**DOI:** 10.3389/fimmu.2024.1359600

**Published:** 2024-03-07

**Authors:** Renuka Ramalingam Manoharan, Ankush Prasad, Pavel Pospíšil, Julia Kzhyshkowska

**Affiliations:** ^1^ Department of Biophysics, Faculty of Science, Palacký University, Olomouc, Czechia; ^2^ Institute of Transfusion Medicine and Immunology, Institute for Innate Immunoscience (MI3), Medical Faculty Mannheim, Heidelberg University, Mannheim, Germany; ^3^ German Red Cross Blood Service Baden-Württemberg - Hessen, Mannheim, Germany; ^4^ Laboratory of Translational Cellular and Molecular Biomedicine, National Research Tomsk State University, Tomsk, Russia; ^5^ Laboratory of Genetic Technologies, Siberian State Medical University, Tomsk, Russia

**Keywords:** oxidative stress, reactive oxygen species, inflammation, innate immune response, macrophage, neutrophils, protein oxidation

## Abstract

The innate immune response represents the first-line of defense against invading pathogens. Reactive oxygen species (ROS) and reactive nitrogen species (RNS) have been implicated in various aspects of innate immune function, which involves respiratory bursts and inflammasome activation. These reactive species widely distributed within the cellular environment are short-lived intermediates that play a vital role in cellular signaling and proliferation and are likely to depend on their subcellular site of formation. NADPH oxidase complex of phagocytes is known to generate superoxide anion radical (O_2_^•−^) that functions as a precursor for antimicrobial hydrogen peroxide (H_2_O_2_) production, and H_2_O_2_ is utilized by myeloperoxidase (MPO) to generate hypochlorous acid (HOCl) that mediates pathogen killing. H_2_O_2_ modulates the expression of redox-responsive transcriptional factors, namely NF-kB, NRF2, and HIF-1, thereby mediating redox-based epigenetic modification. Survival and function of immune cells are under redox control and depend on intracellular and extracellular levels of ROS/RNS. The current review focuses on redox factors involved in the activation of immune response and the role of ROS in oxidative modification of proteins in macrophage polarization and neutrophil function.

## 1 Introduction

**Figure 1:**
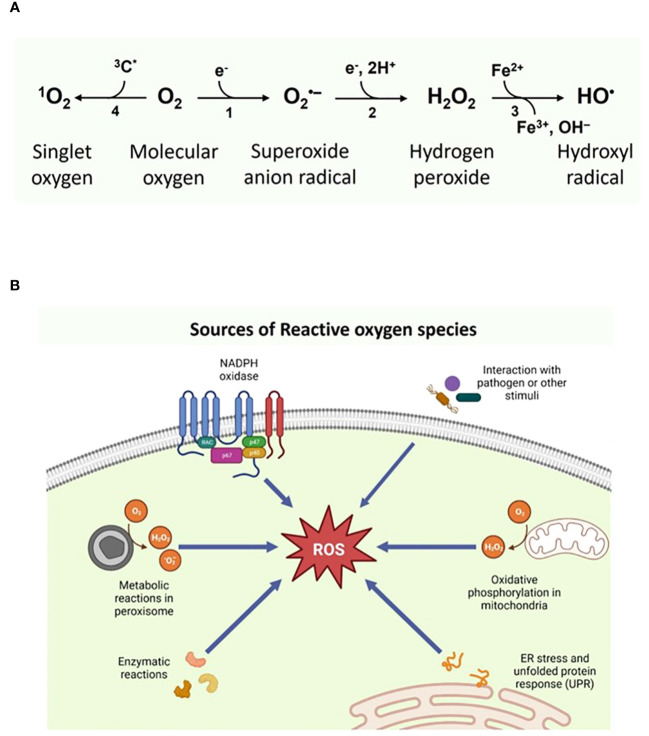
**(A)** Types of Reactive oxygen species and their **(B)** intracellular sources. **Figure 1B** was Created with BioRender.com.

In biology, reactive species formed by redox reaction or electronic excitation hold a growing interest due to their significant impact on the spectrum of pathological processes, including inflammation and aging (1). Based on the nature of reactive atoms, they are named reactive oxygen species (oxygen), reactive nitrogen species (nitrogen) and reactive sulfur species (sulfur), respectively. Produced by nearly all organisms and cells, ROS consists of two subclasses: highly reactive radical and non-radical species. Free oxygen radicals include superoxide anion (O_2_^•-^), hydroxyl (HO^•^), peroxyl (ROO^•^), and alkoxyl (RO^•^), while hydrogen peroxide (H_2_O_2_), singlet oxygen (^1^O_2_), hypochlorite anion and ozone represent the non-radical species (2) (**Figure 1**). ROS subcellular origin and its levels within the cellular environment defines its role. Its function as physiological secondary messengers in signal transduction becoming increasingly apparent; for example, ROS oxidize sulfhydryl (SH) groups of cysteine residues in protein kinases, including protein kinase A(PKA), protein kinase C (PKC), Calcium–calmodulin (CaM)-dependent protein kinase II (CaMKII) and receptor tyrosine kinase (RTK), which activate and phosphorylate their protein targets involved in signaling (3–5).

Reported as the major subspecies within cells, O_2_^•-^ and H_2_O_2_ highly differ in their chemical parameter and functions. An increase in cellular O_2_^•-^ levels remains closely associated with oxidative stress and cellular damage, wherein the oxidation of biological macromolecules and irreversible protein inactivation disturb cellular signaling events (6). H_2_O_2_ is highly diffusible and relatively stable compared to O_2_^•-^ and is considered a pleiotropic physiological signaling agent. In general, H_2_O_2_ at physiological pH mediates signaling via oxidation of cysteine residues, wherein the exposed thiol group (Cys-SH) are deprotonated to the thiolate group (Cys-S^-^) and susceptible to oxidation. H_2_O_2_-dependent signaling occurs with a lower intracellular concentration of approximately 1-100 nM, mediating reversible oxidation of the thiolate group to a sulfenic group (Cys-SOH) and covalent linkage of cysteine residues by disulfide bonds (Cys-S-S-Cys) altering its activity and localization (7). An antioxidant defense system can reverse such protein oxidation and thus serve as essential redox switches in various cellular processes. Nevertheless, excessive production of H_2_O_2_ mediates irreversible nonspecific oxidation of proteins, resulting in a state referred to as oxidative stress (8). This inherent duality of ROS serving as a beneficial secondary messenger in signaling and causing harmful effects through the accumulation of protein adducts signifies its antagonistic pleiotropy (6).

ROS regulate cellular homeostasis and signaling events within a cellular environment, thus serving as primary modulators of cellular dysfunction and contributing to disease pathology. In almost every subcellular organelle, including cytoplasm, endoplasmic reticulum (ER), mitochondria and peroxisomes, ROS are generated as byproducts as a part of its basal metabolic function (9, 10). An imbalance in ROS generation and scavenging by antioxidants results in pathological conditions, including cancer, neurodegenerative disorders and atherosclerosis. Studies carried out so far substantiate the role of ROS in various metabolic processes like mitochondrial ROS promoting monocyte migration, wherein NADPH oxidase (NOX)-mediated cytosolic ROS production aids in innate immunity (11). Depending on cell type, tissue environment and source, ROS participates in normal physiological processes or contribute to metabolic dysfunction and inflammatory signaling in sterile and infectious inflammation.

## 2 ROS in sensing and reaction to damage

### 2.1 ROS in infectious inflammation

During the initial stage of an inflammatory process, immune cells activated in response to invading pathogens or agents release various inflammatory mediators, aiding in increased vascular permeability and leukocyte migration towards the tissue injury site (12). ROS regulates various intracellular adhesion molecules, including ICAM-1, VCAM-1 and selectin expression, ensuring their interaction with leukocytes and transendothelial migration. On the contrary, enhanced expression of superoxide dismutase reduces leukocyte binding to endothelium by decreasing the expression of adhesion molecules (13). Additionally, a gradient increase in H_2_O_2_ at the tissue injury site is an early factor in leukocyte recruitment (14). Cytokines, including tumor necrosis factor (TNF), vascular endothelial growth factor (VEGF), and NOX stimulate cell migration and adhesion (15).

Metabolic reprogramming, which includes changes in the activity of fatty acid oxidation (FAO), tricarboxylic acid (TCA) cycle, glycolysis levels, involvement of pentose phosphate pathway and mitochondrial respiration mediates the phenotypical and functional role of myeloid cells.

Over the past two decades, immune cell functions, namely proliferation and differentiation, have been linked to various metabolic pathways; thus, immunometabolism is inseparable from redox reactions (11). Thus, bioenergetic and biosynthetic demands for T and B cell response, macrophages and dendritic cells are regulated by metabolic pathway and their resulting breakdown products (16). In aerobic organisms, during oxidative phosphorylation in mitochondria, the electron transport chain shuttles electrons to molecular oxygen (O_2_), producing free oxygen radicals. ROS are well known for their role in inflammation through respiratory bursts, wherein macrophages and neutrophils phagocytize pathogens and cellular debris. The process activates the NOX assembly and activation, which triggers further ROS production (17). NOXs play a vital role in inflammatory response through respiratory burst and neutrophil extracellular trap formation. NOX-derived ROS are reportedly involved in angiogenesis, a process of the growth of new vessels and a key event in the proliferative phase of inflammation. It has been reported that NOX remains localized at the leading edge of migrating cells and is linked to actin and IQGAP1 protein (Ras GTPase-activating-like protein), and any disruption at this binding site results in impaired migration of endothelial cells (18).

Nevertheless, enhanced production of reactive species has to be regulated to avoid oxidation of biomolecules leading to cellular toxicity and cell death. Alterations in redox balance are often associated with chronic immune activation, such as autoimmune disorders and neurodegenerative diseases. Therefore, the redox state is an intrinsic cellular and systemic homeostasis indicator.

### 2.2 ROS in sterile inflammation

The release of local damage-associated molecular patterns (DAMPs) in response to tissue damage elicits enhanced cytokine production via sterile inflammation. Cytokines belonging to the interleukin-1(IL-1) family have been proposed to be essential drivers of sterile inflammation, which recruits neutrophils and macrophages to the damaged site. During sterile inflammation, host-derived DAMPs released by stressed cells contribute to macrophage polarization that aids in resolving sterile inflammation and restores homeostasis (19). In endothelial cells, under hyperglycemic conditions, an excessive glucose load triggers ROS formation in mitochondria, impairing its functions and causing cellular damage through interaction with various cellular constituents, including DNA, proteins and lipids (20). These ROS trigger the activation of pro-inflammatory transcription factors, namely NFκB and activating protein-1(AP-1), resulting in enhanced inflammatory cytokines/chemokines expression. In addition, activated endothelial cells attract monocytes that promote inflammation and macrovascular and microvascular injury (21).

Macrophages, especially adipose tissue macrophages (ATM), drive diabetic pathology. In healthy individuals, adipocytes secrete adiponectin, which induces M2-like polarization of ATM and suppresses ROS and its related pathway genes (22). On the contrary, in obesity conditions, a reduction in adiponectin levels induces M1-like polarized macrophages, thereby enhancing glucose consumption through GLUT1 (glucose transporter 1). Thus, an interplay between obesity and hyperglycemia promotes ROS formation, glycolytic metabolism, and the release of pro-inflammatory cytokine mediators by macrophages (23). Hence, focusing more on metabolic reprogramming during macrophage polarization events is essential to identify potential targets for treating inflammation and metabolic disorders. A mitochondrial reactive oxidative stress increase was reported in mice subjected to ventilator-induced lung injury, activating NLRP3 to produce IL-1β and lung inflammation in combination with TLR4 signaling (24).

### 2.3 Sources of ROS production

Though the primary source of ROS *in vivo* is through aerobic respiration, cellular events, including peroxisomal β-oxidation of fatty acids, arginine metabolism, tissue-specific cellular enzymes and phagocytosis stimulation by pathogens also contributed to ROS production (3). Based on its source, cell type and environment, ROS signaling contributes to either normal physiological processes or metabolic dysfunction through inflammatory signaling. Diseased conditions, including diabetes mellitus, atherosclerosis and stroke, are known to be associated with redox balance (25).

The NADPH oxidase (NOX) family of proteins remains the primary cytosolic source of ROS, consist of seven different isoforms and comprises membrane and cytosolic components that are actively involved in the host response to various stimuli including bacterial and viral infections, cellular signaling and regulation of gene expression. Among the isoforms, NOX2 is well characterized for its role in phagocytic functions. Both NOX and inducible nitric oxide synthase (iNOS) are vital in generating enhanced ROS levels within phagocytes via oxidative burst to kill invading pathogens (26). In comparison to mitochondria, O_2_^•-^ produced by NOX is dismutated into H_2_O_2_ by superoxide dismutase1 (SOD1), whereas nitric oxide produced by inducible nitric oxide synthase (iNOS) reacts with O_2_^•-^ resulting in peroxynitrite production (19). For example, ROS drive hypoxia-inducible factor 1α (HIF1α) mediated GLUT1 expression, hexokinase activity, and resultant glycolysis in response to low oxygen tension as part of the angiogenic response (27) The co-localization of neutrophil phosphofructokinase 2 with NOX2 leads to its activation, resulting in NADP+ production as its byproduct, facilitating an enhanced glycolytic rate. The increase in glycolysis rate and enhancement of NOX2 activity and the relation between these processes are still under study (28).

Mitochondria are considered as the redox-active compartment within the cell, accounting for nearly 90% of oxygen utilization (1). They serve as a significant contributor to ROS in the form of O_2_^•-^. Mitochondrial SOD converts O_2_^•-^ into H_2_O_2,_ which in turn gets converted into HO^•^ through the Fenton reaction, which in turn oxidizes biomolecules. SOD1 is constitutively expressed and regulates cytosolic O_2_^•-^ levels (29). Factors like hyperoxia, oxidative stress, and inflammatory cytokines induce SOD2, whereas SOD3 are cell and tissue-specific and likely of significant importance in protection against stress factors from the extracellular environment. In addition, Grx (glutaredoxin), glutathione, and Trx (thioredoxin) systems play a predominant role in mitochondrial ROS buffering, like SOD (30).

Under stress conditions, the ER tubular network holds a unique oxidizing environment, wherein redox signaling mediators play a vital role in ROS generation and mediate protein folding. Protein folding is highly sensitive to ER redox status, and dysregulation of disulfide bond formation in response to ER stress increases luminal oxidative stress, leading to a decline in ER function. During protein folding, protein disulfide isomerase (PDI) and endoplasmic reticulum oxidoreductase 1 (ERO1) introduce disulphide bonds into folded proteins, resulting in H_2_O_2_ formation (31). Protein disulfide isomerase introduces disulfide bonds onto protein substrates through thiol oxidation, resulting in a reduced state. However, PDI is reoxidized through ERO1, which transfers electrons from O_2_ through the flavin adenine dinucleotide cofactor, forming H_2_O_2_ (32). In addition to the PD1/ERO1 pathway, ROS are produced through NOX4, NADPH-P450 reductase (NPR), and GSH (33). NOX4 is reported to be consistently associated with ER, and NOX4 associated with p22phox utilizes NADH or NADPH as an electron donor to produce O_2_^•-^. They are also reported to interact with PDI, whereas the absence of PDI results in cell death (34). In macrophages, the interaction between p22phox and PDI was observed (26).

Peroxisomes, like mitochondria, are vital organelles that regulate crucial processes such as α- and β-oxidation, amino acid catabolism, glyoxylate metabolism, ketogenesis, polyamine oxidation and isoprenoid and cholesterol metabolism (35). Peroxisomal electron transfer leads to free electrons rather than ATP, which are transferred to H_2_O to form H_2_O_2_. In peroxisomes, H_2_O_2_ is produced by various oxygen-consuming oxidases, including D-amino acid oxidase, xanthine oxidase, d-aspartate oxidase, polyamine oxidase and acyl-CoA oxidase. In addition, peroxisomal oxidases and xanthine oxidases generate ROS and nitric oxide (36). The lysosomal electron transport chain generates HO^•^ via proton translocation to maintain an optimal pH for acidic hydrolases (9).

Thus, ROS are produced as a byproduct of cellular events wherein the NOX family of proteins mediate the reduction of O_2_ to O_2_^•-^ and phagocytes NOX accounts for an increased amount of O_2_^•-^ and H_2_O_2_ production by respiratory burst (3). Upon stimulation, the membrane-bound catalytic core assembles with proteins from the cytosol (p47 phox, p67 phox and small G protein Rac), activating O_2_^•-^ production. ^•^NO produced by Nitric oxide synthase (NOS) can migrate through the cell membrane via diffusion and mediate several signaling pathways in a dose-dependent manner (37). However, inflammatory activation of iNOS by cytokines or lipopolysaccharides enhances cellular levels of ^•^NO and results in inflammatory diseases and septic shock (38). Both oxidative and nitrosative stress can hinder the functioning of intracellular redox buffer systems, resulting in decreased antioxidant capacity of affected cells (39). Thus, a proper balance between ROS-RNS is essential in regulating immunological response. A schematic representation of types and sources of ROS are presented in **Figures 1A, B**, respectively.

### 2.4 Mechanisms of oxidative protein modifications

#### 2.4.1 Oxidation of sulfur-containing and aromatic amino acids

Sulfur-containing biomolecules are crucial in protein folding, deactivation of reactive species, enzymes, redox signaling and other biochemical functions. Remarkably, most of the functions are associated with proteins and protein adducts, whereas its functions can be traced back to two amino acids, cysteine and methionine and their respective thiol or thioether functionality (40, 41). Oxidative stress targets the sulfhydryl group of cysteine and the methionine thioether group, resulting in increased post-translational modification events. Being sensitive to redox transformations, thiol, the side chain of cysteine, acquires different oxidation states. While thiol and disulfide are commonly known, growing evidence of protein modification also reports other oxygen derivatives, including sulfenic, sulfinic and sulfonic derivatives. Disulfide formation remains the most common thiol oxidation wherein the disulfide bonds are reasonably stable and stabilize protein structures via intra- and intermolecular disulfide bridges. Cysteine thiyl radical and sulfenic acid formation is reversible, and both intermediates are highly unstable. Both serve as precursors for several oxidized cysteine modifications (42). Sulfenic acids are highly reactive and play a prominent role in enzyme catalysis and cell signaling. They remain as key intermediates to other oxidation states, namely sulfinic and sulfonic acids. During the inflammatory process, immune cells, thiol or thiolate anion reaction with hypochlorous acid (HOCl) result in the formation of sulfenic acids (43).

Approximately 5% of cellular proteins remain in either sulfinic or sulfonic acid forms. The functional role of sulfinic acid modification has been reported mainly with the peroxiredoxin (Prxs) family (44), which reduces H_2_O_2_ and alkyl peroxides to water and alcohol. Under physiological conditions, in contrast to sulfenic acids, sulfinic derivatives do not react with thiols or undergo self-condensation reactions. Sulfinic acids are stable intermediates but oxidize readily to sulfonic acid (RSO3H), the most highly oxidized species of thiols and disulfides. Potent oxidizing agents, halogens, H_2_O_2_, and nitric acid can generate sulfonic acids from thiols (20). Introducing highly oxidized sulfur species can result in protein structural changes or inhibit enzyme activity that requires thiolate for catalysis. Alternatively, these reported cysteine oxidation products also serve as a prerequisite for proper protein function. Thus, the irreversible oxidation of cysteine to sulfinic and sulfonic acid can influence cellular homeostasis and protein functions in multiple ways. Oxidation of cysteine to sulfenic and sulfinic acid modifications can be reversed by S-glutathionylation, wherein glutaredoxin mediates sulfinic acid reduction, conjugation of sulfenic acid via *S*-glutathionylation, and deglutathionylation by glutaredoxin or sulfiredoxin. As noted by the N-end rule pathway, irreversible cysteine oxidation can also target a protein for degradation; for example, oxidation of N-terminal cysteine residues to sulfinic and sulfonic acid in specific mammalian proteins, such as GTPase-activating proteins (RGS) is required for arginylation by ATE1 R-transferases and subsequent ubiquitin-dependent degradation. Thus, the overoxidation of cysteine to sulfonic acid cannot be reversed, and the damaged proteins have to be degraded by the proteasome (45). Apart from cysteine, the other sulfur-containing amino acids that undergo oxidative modification include methionine, which is reduced to methionine sulfoxide by methionine sulfoxide reductases. Methionine sulfone formation resulting from further oxidation events is considered a stable modification.

Concerning aromatic amino acids, tyrosine remains the primary target of protein oxidation events due to its redox-active structure. Its phenolic side chain gets oxidized easily, forming an intermediary tyrosyl radical. Upon reaction with HO^•^, these radicals form 3-hydroxylysine, a neurotransmitter analogue 3,4 dihydroxyphenylalanine (DOPA) and, in interaction with another tyrosyl radical, forms a fluorescent protein crosslink dityrosine. Hydroxyl radicals, upon interaction with tryptophan and histidine, form hydroxytryptophan and 2-oxohistidine, respectively (46).

#### 2.4.2 Glycoxidation

Reported as a spontaneous non-enzymatic reaction, glycation involves the response of free-reducing sugars with lysine and arginine amino acid residues, DNA and lipids forming Amadori products. This product, in turn, undergoes irreversible rearrangement and dehydration reaction, leading to the formation of advanced glycation end products (AGEs) (47). Introduced by Louis-Camille Maillard in 1912, glycation results in loss of protein function and impaired tissue elasticity in the skin, blood vessels and tendons. Glycation reactions are reported to be enhanced during oxidative stress and hyperglycemia conditions, thus playing a pivotal role in the pathogenesis of diabetic complications and aging. The formation of AGEs does not entirely rely on oxidative conditions; more specifically, only selected AGEs are generated by oxidation. Formed by a combination of glycation and oxidation, a subset population of AGEs is termed glycoxidation products. Excessive generation of ROS from glucose autoxidation and covalent attachment of glucose molecules to circulating proteins results in the formation of AGEs (48). They serve as biomarkers for both oxidative and carbonyl stress. Carboxymethyl lysine (CML), reported as the most abundant AGEs *in vivo*, is formed by oxidative degradation of Amadori product fructoselysine. An alternative non-oxidative mechanism involves the reaction of α-dicarbonyl compound glyoxal and lysine, leading to CML formation via an isomerization mechanism. The oxidative degradation of carbohydrates, lipids, nucleotides, and serine mainly forms the precursor, glyoxal. Further oxidation reaction of this glyoxal results in the formation of α-oxoamide AGE glyoxylyl lysine. As it relies on oxidative processes, glyoxylyl lysine is considered an even more sensitive marker than CML. Apart from oxidatively formed glycoxidation products, some AGEs are formed by precursors generated by oxidation, for example, glucose oxidation or Amadori products to glucosone. Lysine-mediated cleavage of glucosone results in formyl lysine formation, and reports from recent studies confirm high levels of formaldehyde metabolism products (formyl lysine, formyl phosphate) in murine tissues (49).

#### 2.4.3 Lipid peroxidation

An increase in levels of ROS can impose direct damage to lipids. The most prevalent ROS reported to affect lipids profoundly include HO^•^ and hydroperoxyl (HO_2_^•^). In biological systems, the Fenton reaction forms HO^•^ through redox cycling. Hydroperoxyl radicals play an essential role in lipid peroxidation, wherein this protonated form of O_2_^•-^ produces H_2_O_2_, which can react with redox-active metals, yielding HO^•^. Hydroperoxyl radicals (HO_2_^•^) are reported to be much stronger than O_2_^•-^ and could initiate the oxidation of polyunsaturated phospholipids, thereby impairing membrane function (28). The lipid peroxidation process involves hydrogen abstraction from carbon with oxygen insertion, resulting in lipid peroxyl radicals and hydroperoxides (LOOH). More specifically, free oxygen radicals target lipids containing carbon-carbon double bond(s), especially polyunsaturated fatty acids (PUFAs). Under physiological or subtoxic lipid peroxidation rates, cells survive by upregulation of antioxidant pathways and proteins, whereas, at toxic concentration levels, cells induce apoptosis, eventually leading to cellular damage (32). Thus, lipid peroxidation events might facilitate disease progression and aging. During the process of lipid oxidation, several reactive carbonyl species (RCS) are formed, which include LOOH and different aldehydes formed as secondary products, such as malondialdehyde (MDA), propanal, hexanal, and 4-hydroxynonenal (4-HNE) (4).

Malondialdehyde is reported to be the most abundant secondary aldehyde generated by the decomposition of arachidonic acid and larger PUFAs. The reactivity of MDA is pH-dependent; thus, at physiological pH, it exists as an enolate ion with low reactivity. Upon a pH decrease, MDA enolizes to β-hydroxy acrolein with increased reactivity. Malondialdehyde initial reaction with proteins generates Schiff-base adducts referred to as advanced lipid peroxidation end-products (ALEs). Under oxidative stress conditions, acetaldehyde in the presence of MDA generates highly immunogenic malondialdehyde acetaldehyde (MAA) adducts. These adducts are of biological importance as they can alter the functional properties of biomolecules, resulting in disease progression. Protein Kinase C (PKC) plays a vital role in the intracellular signal transduction process, which involves cell proliferation and differentiation, inflammation and cytoskeletal organization. The binding of MAA adducts induces activation of PKC-α, a specific isoform in hepatic cells, resulting in increased secretion of urokinase-type plasminogen activator, causative of hepatic fibrosis (44). Another important example of α/β unsaturated RCS is 4-hydroxy-2- nonenal (4-HNE). They are reported to be highly reactive, wherein nucleophilic attack of cysteine and histidine forms stable Michael adducts (45). 4-HNE protein adducts can contribute to protein crosslinking and induce carbonyl stress. For example, 4-HNE is reported to modify membrane-associated protein, G-protein signaling 4 (RGS4) at cysteine residue during oxidative stress, thereby altering signaling events in stressed cells (46). However, based on their cellular level and pathways involved in lipid peroxidation products, MDA and 4-HNE pose a dual behaviour of either enhancing cell survival or promoting cell death.

#### 2.4.4 Protein carbonylation

ROS-mediated protein carbonylation events are characterized as the most common type of non-enzymatic post-translational modification (PTM). This stable modification is achieved by either direct oxidation of protein-bound amino acids, oxidative cleavage of the protein backbone and incorporation of carbonyls from glycoxidation or lipoxidation. ROS/reactive intermediates such as H_2_O_2_ and lipid hydroperoxides interact with specific amino acids, arginine, lysine, proline or threonine, causing protein-protein cross-linkages, resulting in protein denaturation and loss of activity. Various oxidation products have been reported so far, which include tryptophan forms kynurenine, nitrotryptophan; Phenylalanine forms 2,3- 2-, 3-, and 4-hydroxyphenylalanine, Dihydroxyphenylalanine; Histidine forms 2-Oxohistidine; Arginine and proline forms glutamic semialdehyde (50). Superoxide anion radical formation from O_2_ or HO^•^ by the interaction of H_2_O_2_ with free iron (Fe^2+^) through the Fenton reaction results in the interaction of ROS with the amino acids mentioned above. Superoxide anion radical generated from O_2_ is converted by SOD to H_2_O_2_ and later into H_2_O by catalase, glutathione peroxidase or peroxiredoxin (47). The direct oxidation of amino acids, aminoadipic and glutamic semialdehyde contribute to approximately 60% of total protein carbonylation in the liver. Hydroxyl radical-mediated abstraction of hydrogen located next to the N6-amino function of lysine, metal-catalyzed oxidation of the carbon-centered radical, and hydrolysis of the resulting imine mediates aminoadipic semialdehyde formation. Concerning oxidative cleavage of the protein backbone, O_2_^•-^ facilitates RO^•^ formation at α-carbon next to a peptide bond. The RO^•^ fragments either through the diamide pathway (homolytic cleavage of carbon-carbon bond) or the α-amidation pathway (carbon-nitrogen bond) (50). Protein carbonylation remains a valuable biomarker in aging and diseases, wherein they are shown to impair protein structure and function. In a carbonylated protein profiling study from lean and obese individuals with or without type 2 diabetes (T2D), 36 out of 158 unique carbonylated proteins were reported to be present only in obese patients with T2D. These identified proteins were found to play a vital role in intracellular signaling and angiogenesis, cell adhesion and cytoskeletal remodeling (51).

Highly oxidized proteins appear to be relatively poor substrates for degradation by ubiquitination. Thus, dysfunctional carbonylated proteins accumulate as covalently crossed protein aggregates, making them highly resistant to proteolysis, thereby affecting the functional integrity of cells during the aging and disease processes. On the contrary, proteins that have undergone mild oxidation are highly susceptible to proteasomal degradation due to exposure to hydrophobic amino acids by unfolding targeted protein domains. Hydrophobic surface patches remain the central motif recognized by the proteasome. Remarkably, 19S and 20S proteasome subunits are highly susceptible to carbonylation and HNE modification, suppressing their proteolytic activities (52). Protein carbonylation may also be beneficial by regulating and activating signaling pathways involved in antioxidant defense and cellular homeostasis. Carbonylation depends on the cellular redox environment, ROS abundance and its proximity to the proteins.

#### 2.4.5 Nitrosylation

Nitric oxide (NO), also referred to as Nitric oxide radical (^•^NO), remains an important signaling molecule that exhibits pleiotropic functions like vasodilation, neurotransmission and pro-inflammatory signaling. Nitric oxide synthase (NOS) utilizes L-arginine as a substrate and, along with oxygen, produces citrulline and NO. They are reported to mediate both anti- and pro-oxidant mechanisms. In immune cells, nitric oxide limits ROS production via NADPH oxidase and pretreatment of cells with NO protects them against oxidative stress. Recent clinical trials support the beneficial effects of NO pretreatment in ischemia–reperfusion-mediated tissue injury (53). On the contrary, NO readily reacts with O_2_^•-^ forming RNS, peroxynitrite (ONOO^−^) and nitrogen dioxide (NO_2_). *In vivo*, these RNS are reported to be potent oxidizing agents that can directly or reversibly modify cysteine residues through *S*-nitrosylation. Nitrogen dioxide radicals interact at the ortho-position of the tyrosine aromatic ring, resulting in the formation of irreversible modification of 3-nitrotyrosine. Both peroxynitrite and protein tyrosine nitration is reportedly involved in aging. Protein tyrosine nitration serves as a potential biomarker of disease progression. While initial experiments demonstrated the involvement of endogenous peroxynitrite and protein tyrosine nitration in apoptosis of motoneurons in culture, further work in ALS animal models confirmed the formation of 3-nitrotyrosine and protein-derived radicals in spinal cord motor neurons during disease progression (54, 55). Various protein oxidation events used as biomarkers in clinical settings are presented in **Table 1**.

**Table 1:** Oxidative modification of proteins as biomarkers in clinical studies.

Type of oxidative modification	Pathway	Role of ROS	Cells	Biological significance	Reference
Carbonylation	Direct oxidation of amino acids/ Cleavage of peptide bonds by α-amidation pathway	Formation of alkoxyl radical by superoxide	Activated platelets, adipocytes, hippocampus and neocortex in AD and Lewy bodies of PD	Elevated levels of protein carbonyl levels in T2D patients with vascular complications; Enhanced protein carbonyl levels in RA and HIV patients; In comparison to PD, higher level protein carbonyls were reported in AD patients.	(56–60)
Lipid peroxidation	Non-enzymatic autooxidation /Enzymatic phospholipid peroxidation	radical (HO^•^) and non-radical (^1^O_2_) reaction	Mitochondria, plasma membrane, and peroxisomes	Higher levels of 4-HNE in patients with T2D; Elevated levels of free and protein-bound 4-HNE in RA Patients; In comparison to control subjects, elevated levels of MDA reported in T2D patients with and without chronic kidney diseases; In AD and PD patients, lesser levels of MDA were reported compared to control subjects; Higher levels of MDA in the elderly population;	(61–63)
Glycoxidation	Amino acids lysine, cysteine and histidine undergo carbonylation (formation of advanced glycated end products AGEs) upon interaction with reactive carbonyl group produced by carbohydrate oxidation	hydroxyl radicals (HO^•^) interact with sugars and produce initially a secondary hydroxyl alcohol radical which is further oxidized with O_2_ to hydroxyalkyl peroxyl radical	Endoplasmic reticulum	Higher levels of AGEs in T2D patients’ serum with or without vascular complication; Elevated levels of protein-bound CML were observed in AD and PD patients in comparison to healthy populations.	(64–66)
Nitration	Addition of nitro-(NO_2_) group to ortho position of tyrosine residues in proteins.	Nitric oxide synthase (NOS) utilizes arginine and oxygen to produce ^•^NO and citrulline. ^•^NO reacts with superoxide anions resulting in the formation of RNS namely peroxynitrite and nitrogen dioxide	Mitochondria	Higher levels of protein-bound 3NT were reported in patients with T2D; Oral squamous cell carcinoma tissue samples with metastases exhibited higher 3-NT levels in comparison to samples without metastases.	(67–69)

## 3 Redox factors involved in immune cell activation

### 3.1 Redox factors in macrophage activation and function

Macrophages are a heterogeneous population of immune cells that play a vital role in tissue homeostasis in response to pathogen infection by phagocytosis and mediate tissue repair during injury. They rapidly recognize, engulf and destroy pathogens or apoptotic cells, which can be attributed to their plasticity and heterogeneity (70). Through polarization events, macrophages adopt either a pro-inflammatory phenotype classified as M1 macrophage or an anti-inflammatory M2 phenotype that mediates wound healing and inflammation resolution (71). Recently, it has been observed that excess molecular stimuli induce diverse and partially overlapping macrophage phenotypes that are distinct from M1 and M2. Macrophage activation by ROS, cytokines and commensal lipopolysaccharide (LPS) results in the activation of NF-κB and PI3K/AKT signaling pathways (72, 73). Thus, upregulated NF-κB increases pro-inflammatory chemokines and cytokine transcription, inducible NO synthase (iNOS) and HIF1 α. These signaling pathways, enzymes, and transcription factors are essential in maintaining macrophage activation and M1 polarization by driving metabolic reprogramming (17).

**Figure 2:**
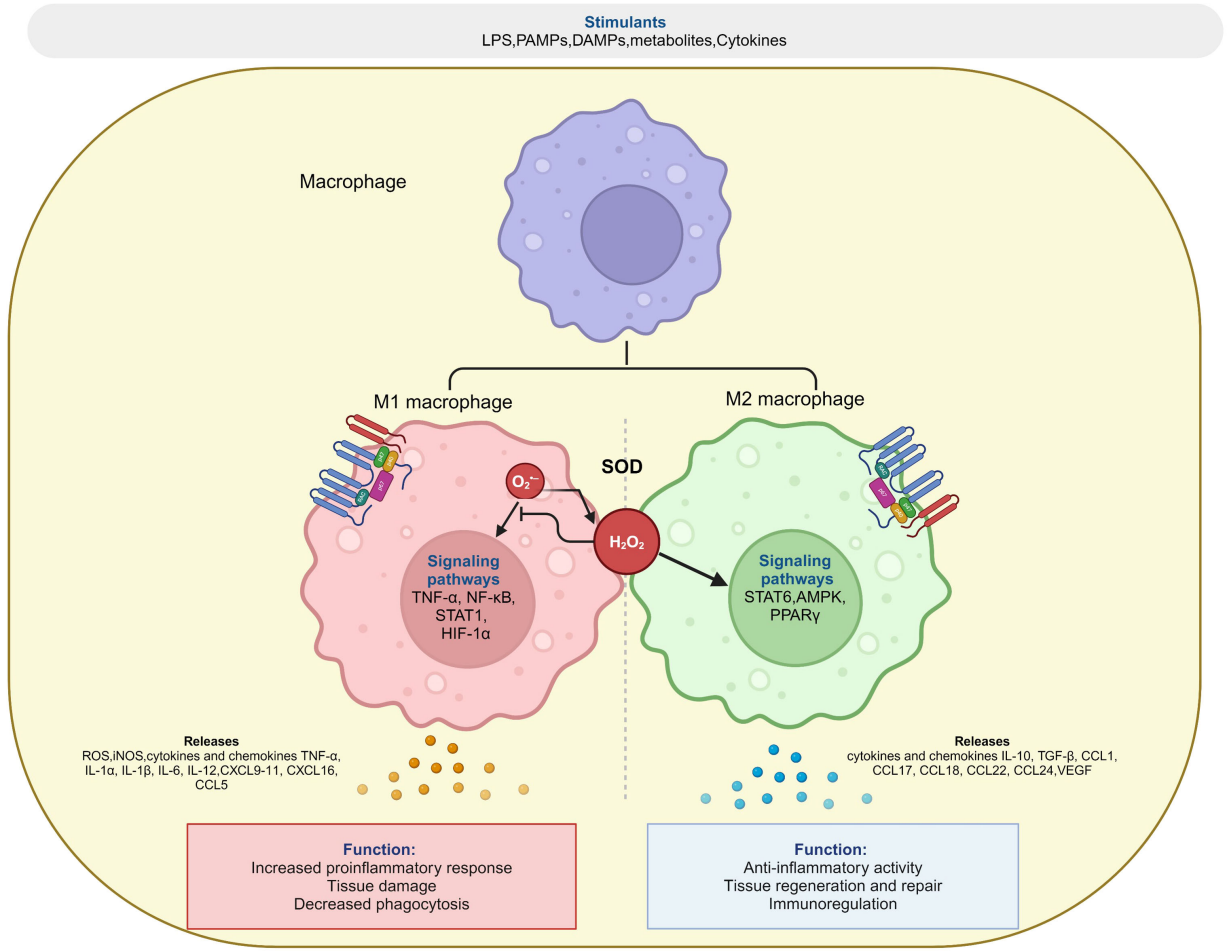
Redox regulation of macrophage polarization. Superoxide (O_2_^•-)^ generated by NOX or mitochondrial electron transfer chain is converted into H_2_O_2_ by superoxide dismutase (SOD), which balance between both pro-inflammatory (M1) and anti-inflammatory (M2) response of macrophage during polarization events. The figure was Created with BioRender.com.

ROS regulate the intracellular signalosome within a constantly evolving cellular microenvironment, thus underlying its role in polarization and the specialized function of immune cell populations (74). M1 and M2 macrophages differ from resting macrophages in their phenotype and exhibit distinct metabolic profiles (75). M1 macrophage metabolism is characterized by aerobic glycolysis, changes in pentose phosphate pathway (PPP), FAS, and truncated TCA cycle, while M2 mostly depends on oxidative metabolism, fatty acid oxidation (FAO) and decreased glycolysis (76) (**Figure 2**).

### 3.2 Metabolic reprogramming in M1 macrophages

Glycolysis is a crucial metabolic event for M1 macrophages, and its inhibition affects typical functions of their inflammatory phenotype, including phagocytosis, ROS production, and pro-inflammatory cytokine secretion. Lipopolysaccharides and Toll-like receptor (TLRs) mediated differentiation of M1 macrophages are associated with a metabolic shift towards glycolysis, blocking it by glucose derivate like 2-deoxyglucose impairs both ROS and pro-inflammatory cytokine production. Numerous transcription factors are known to be involved in maintaining metabolic changes associated with M1 macrophages, and notable factors were discussed in detail. Reports demonstrated the involvement of HIF1α in activating inflammatory macrophage through the glycolysis mechanism (77). They act as modulators of the methylation status of hypoxia-responsive elements in the promoter regions. Enhanced expression of HIF1α reduces mitochondrial activity by suppressing electron transport chain enzymes, resulting in mitochondrial autophagy. HIF1α exaggerates glycolytic flux, thereby increasing the expression of glucose transporters (GLUT1 and GLUT3) and inflammatory mediators. More specifically, M1 macrophages rely on glycolysis and accumulation of succinate from the TCA cycle to stabilize HIF1α, which in turn activates the transcription of glycolytic genes sustaining glycolytic metabolism in M1 macrophages (36). Increased levels of HIF1α were evident along the differentiation of monocytes into tissue macrophages and play a prominent role in the uptake of bacteria by macrophages under hypoxic conditions and expression of tumour necrosis factor (TNFα) and nitric oxide (NO) through inducible NO synthetase (iNOS). In macrophages, overexpression of glucose receptors enhances glycolysis, which induces ROS production and pro-inflammatory mediators (78). In M1 macrophages, aerobic glycolysis induction depends entirely on redox-sensitive transcription factor HIF1α activated by the NF-κB pathway during inflammation. HIF1α interacts with pyruvate kinase isoenzyme M2 (PKM2), thereby mediating the transcription of glycolytic enzymes and inflammatory factors like IL-1β. ROS-like NO-mediated prolyl hydroxylases (PHDs) inhibition induces HIF1α (79). Further, NO reduces oxidative phosphorylation by nitrosylation, inhibiting proteins involved in the mitochondrial electron transport chain. Thus, ROS production positively regulates and maintains the shift towards aerobic glycolysis in M1 macrophages.

NADPH generated by PPP regulates the inflammatory response of M1 macrophages. They mediate ROS and NO production through NOX and iNOS, respectively, to kill invading pathogens and sustain the functionality of TRX and GSH antioxidant systems. Results presented by Nguyen and co-workers demonstrate that deletion of the TRX1 system impairs NLRP3 inflammasome formation and binding of NF- κB to target DNA in monocytes and macrophages (10). It leads to defective production of pro-inflammatory cytokines and ROS accumulation. Additionally, mitochondrial ROS-induced DNA damage aid in a significant drop in NAD+ levels in M1 macrophages. Suppression of PPP in macrophages attenuates LPS-induced inflammatory and oxidative stress response. Compared to glycolysis, intermediates of the TCA cycle, namely succinate and citrate, support biosynthesis in M1 macrophages (11). In LPS-stimulated macrophages, two breaks in the TCA cycle result in the accumulation of succinate and citrate, stabilizing HIF-1α and the subsequent increase in IL-1β transcription. Furthermore, succinate dehydrogenase-mediated oxidation of succinate and increased mitochondrial membrane potential drive ROS production (72). Another TCA cycle metabolite, citrate, gets transported into the cytosol and utilized for fatty acid synthesis to support membrane biogenesis and synthesis of pro-inflammatory lipid mediators, namely prostaglandins. In LPS-activated macrophages, ROS-dependent oxidation of unsaturated phospholipids results in glutamine utilization to feed the TCA cycle and lead to cytoplasmic accumulation of oxaloacetate. These metabolites stabilize HIF-1α, enhancing IL-1β secretion in atherosclerosis (13).

In M1 macrophages, ROS-mediated activation of NRF2 is essential for PPP maintenance and NADPH production, which is necessary for FAS, TRX, and GSH systems. IL-1β is produced as an inactive precursor in response to pathogens, and thus, further processing of it into a biologically active form requires the formation of multiprotein complexes termed inflammasomes. Mitochondrial ROS and its oxidation products are known to play a prominent role in inflammasome activation, whereas the role of NOX is highly dispensable (14).

### 3.3 Metabolic reprogramming in M2 macrophages

Unlike M1 macrophages, M2 macrophages hold an intact TCA cycle and enhanced mitochondrial OXPHOS. CD36 internalizes circulating lipoproteins and fatty acids, mediating fatty acid uptake and fueling OXPHOS. The increased cellular concentration of IL-4 and IL-13 drives M1 macrophages towards anti-inflammatory and healing phenotypes described as M2 macrophages. Tyrosine phosphorylation and signal transducer and activator of transcription 6 (STAT6) activation mediate polarization of macrophages into the M2 phase. IL-4 and IL-13 suppress pro-inflammatory cytokine production by upregulating transforming growth factor beta (TGF-β) activity (70). Adenosine 5′-monophosphate-activated protein kinase (AMPK) and peroxisome proliferator-activated receptor (PPAR) are found to play a vital role in the transition of macrophage polarization states through IL-13 and IL-4. AMPK inhibits NF-κB and stimulates OXPHOS and FAO, reducing HIF1α levels and inflammation and terminating aerobic glycolysis (80). AMPK negatively regulates LPS-induced inflammatory response in macrophages by inhibiting NF-κB activity and activating the PI3K/Akt signaling pathway. Enhanced IL-10 expression promotes TAM activation by PPARα/β, resulting in a polarization of M2 macrophages (12). The importance of FAO in M2 polarization was highlighted in several studies wherein blocking FAO with inhibitors against mitochondrial carnitine palmitoyl-transferase 1 inhibited the activation of M2 macrophages. Results from various studies confirm the PPARγ-mediated activation of M2 signature genes either through oleic acid and IL-4 stimulation or by promoting glutamine oxidation fueling OXPHOS (81). Modification in arginine metabolism emphasizes the transition from M1 to M2 polarization. Increased activity of iNOS mediates arginine metabolism to produce NO, which maintains the switch towards aerobic glycolysis in M1 macrophages. In the case of M2 macrophages, arginine gets metabolized into ornithine and urea due to increased transcription of arginase-1. Both urea and ornithine are essential in M2 macrophage proliferation and survival. Additionally, glutamine metabolism is particularly interesting since glutamine oxidation depletes extracellular glucose levels in an inflammatory environment, maintaining TCA activity and activating the glutamine–UDP-N-acetylglucosamine (GlcNAc) pathway to reinforce M2 polarization (82).

### 3.4 Redox regulation of neutrophil activation

**Figure 3:**
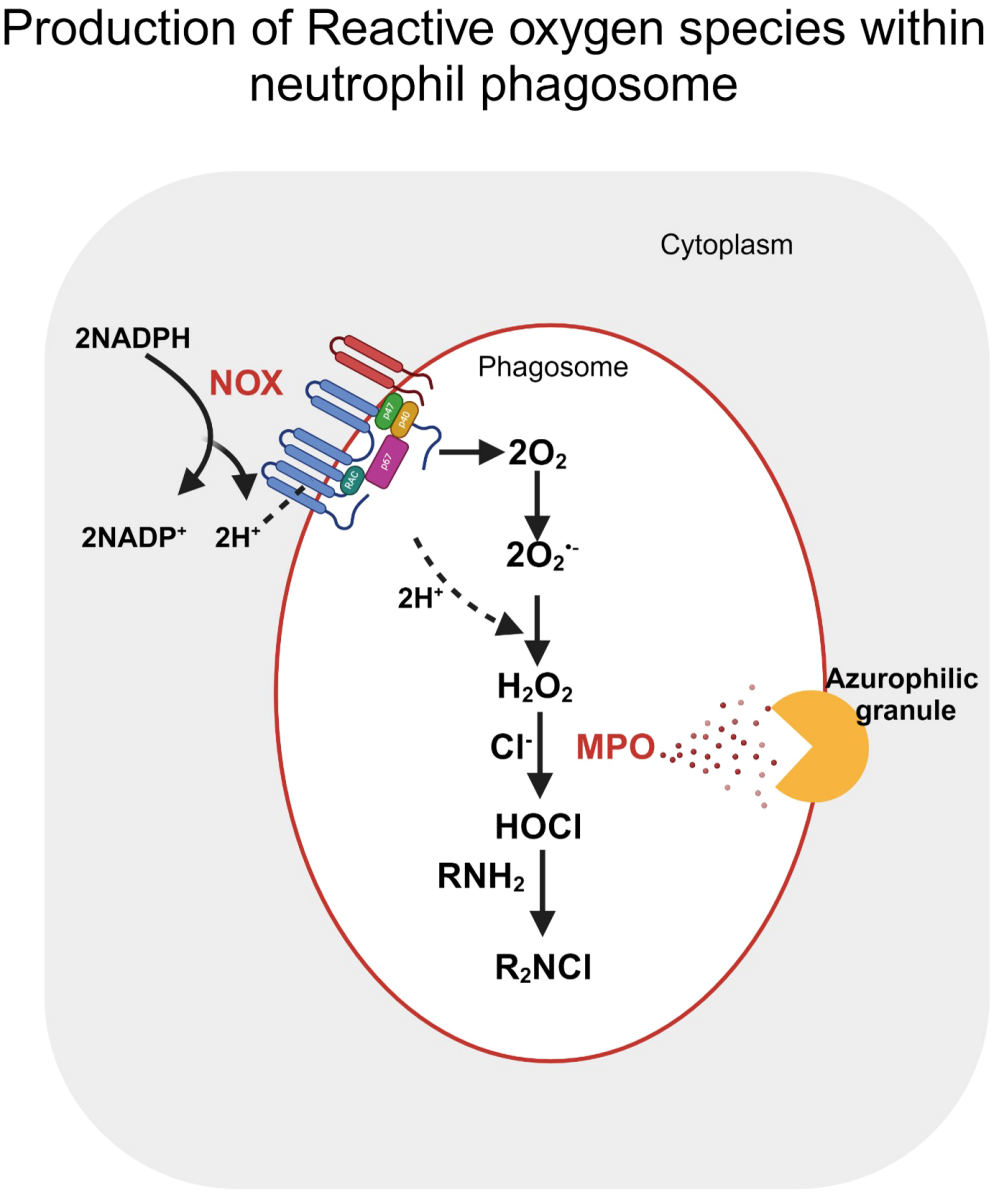
Reactive oxygen species production within the neutrophil phagosome. Post stimulation, oxygen reduction by NOX in the presence of NADPH produces superoxide (O_2_^•-)^ within the phagosome. H_2_O_2_ produced by either the enzymatic or spontaneous dismutation of Superoxide further stimulates neutrophil granules, releasing myeloperoxidase (MPO) within the phagosome. MPO catalyses the oxidation of halides (Cl-) by interaction with H2O2, forming HOCl. The figure was Created with BioRender.com.

Neutrophils are the first responders against invading pathogens through innate and humoral immunity. Activated neutrophils rely on glycolysis as their primary source of energy under both physiological and inflammatory environments; however, they can regulate their metabolism to carry out its effector functions, namely phagocytosis, oxidative burst, degranulation, extracellular trap formation and chemotaxis (83). Neutrophil extracellular trap (NETs) formation by neutrophils relies on glycolysis and PPP as a source of NADPH, resulting in free oxygen radical production. Superoxide anion radical, thus produced, further induces the formation of ROS and HOCl used by neutrophils in oxidative bursts following phagocytosis of invading pathogens. ROS promote several steps of NETosis, including releasing neutrophil elastase from granules by increasing membrane permeability and degradation of H1 linker and core histones resulting in chromatin decondensation (84). The morphological changes associated with NETosis were promoted by ROS, which in turn inactivated caspases to block apoptosis and trigger autophagy. Secondary oxidants, namely HOCl, mediate PMA-induced NETosis, and it entirely depends on NOX activity (**Figure 3**). The absence of extracellular Cl^-^, a substrate for Myeloperoxidase (MPO) *in vitro* results in decreased NET production (85). On the contrary, calcium ionophores induced NETosis are NOX independent and rely on mtROS. NOX-independent Netosis depends on calcium which in turn activates peptidyl arginase deiminases resulting in cellular hypercitrullination (86).

Neutrophil functions are highly influenced by cellular redox status, which includes both ROS/RNS production and cellular antioxidant systems (87). Enhanced ROS production is reported to comprise phagocytosis, resulting in dysregulated oxidative burst events and NET production. ROS levels determine the sensing of pathogens by neutrophils and their subsequent activation of NLRP3 inflammasome and cytokine synthesis (88). Additionally, chronically upregulated ROS and cytokine production lowers neutrophil migration by internalizing CXCR2, a membrane chemokine receptor. Neutrophil functions, namely oxidative burst and NET formation, were sustained by the glutathione system (GSH). Its basal activity was reported to be lower in neutrophils compared to other myeloid cells. Prolonged neutrophil activity and excessive production of MPO due to chronic nitrooxidative stress and inflammation lead to the depletion in GSH levels (89). Thus, depleted GSH levels in neutrophils affect their chemotaxis, transmigration and cytoskeletal reorganization, resulting in early apoptosis and impaired degranulation. Redox factors involved in macrophage and neutrophil function are presented in **Table 2**.

**Table 2:** Redox mechanism influencing macrophage and neutrophil functions.

Redox mechanism	Macrophage function	Neutrophil function	Reference
Reactive oxygen species (ROS)/ Reactive nitrogen species (RNS)	Serve as signaling molecule thereby enhancing inflammatory signalling via NF-KB and MAPK; Modulates NADPH oxidase assembly thereby increasing superoxide and peroxynitrite formation.	Sensing of pathogen; Dysregulate oxidative burst and NETprodcution; Compromises phagocytosis; NO and Peroxynitrite compromise neutrophil migration and adhesion	(14); (89, 90)
Glutathione (GSH) and GSH reductase	GSH protect against oxidative damage by acting as a scavenger; Regulates pro-inflammatory status (M1) of macrophage.	GSH reductase withstands neutrophil respiratory burst and NET production	(16, 91)
Nuclear factor erythroid 2-related factor 2 (Nrf-2)	Mediates anti-inflammatory effects by attenuating IL-6 and IL-4	Neutrophil phagocytosis	(16, 92)
Thioredoxin (TRX)	Promotes binding of NF- κB to DNA and NLRP3 inflammasome; Reduces inflammation and favors M2 polarization	Neutrophil chemotaxis; desensitization of neutrophils toward monocyte chemoattractant protein-1	(93, 94)

### 3.5 Immune cells oxidants mediated epigenetic regulation

Multiple transcriptional and epigenetic modification factors are known to be involved in macrophage differentiation and its activation. Epigenetic changes within macrophages allow them to switch between cellular programs affecting phenotype plasticity. DNA demethylation is known to be involved in the process of monocyte-to-macrophage differentiation. DNA demethylation affects specific genes that regulate actin cytoskeleton and phagocytosis (19). ROS-mediated oxidation of amino acid residues in histone H3 results in chromatin relaxation and accumulation of transcription factors. Cysteine residues at histone H3 sense redox changes and mediate further opening of chromatin structures (95). In LPS-stimulated macrophages, lipid peroxidation products are reported to form lysine adducts with H2, H3 and H4, including H3K23 and H3K27. These modifications at histone acetylation and methylation sites are known to be associated with the epigenetic patterning of cardiovascular diseases (96). LPS stimulation and TLR-4-dependent activation of inflammatory genes primarily depend on H3 and H4 acetylation. ROS-mediated post-translational modification of both class I and II histone deacetylases impair its enzymatic function, resulting in open chromatin structure.

Additionally, lipid peroxidation products mediate the carbonylation of HDACs, resulting in ubiquitination and proteasomal degradation of HDAC function, increased acetylation of histones in macrophages and release of pro-inflammatory cytokines. HDACs are predominant in regulating immunological pathways, more precisely in M1 activation. The difference in the expression pattern of almost all HDAC classes was observed in cells stimulated with LPS. Studies concerning macrophage stimulation with LPS result in an initial decrease in HDAC 4,5 and 7 expression, thereby leading to cyclooxygenase-2 gene activation (97). On the contrary, HDAC6 aids in the expression of pro-inflammatory genes in macrophages stimulated with LPS and thus, its inhibition limits macrophage activation. Concerning the link between DNA methylation and LPS stimulation, SOCS1, a negative regulator of cytokine signals, has been found. DNMT1-mediated hypermethylation of SOCS1 results in the loss of its activity, thereby enhancing the expression of LPS-induced Pro-inflammatory cytokines, namely TNF-α and IL-6. DNMT1 has also been reported to improve the demethylation and trimethylation events of H3K9 in regulator proteins like Notch1 and KIF4 and mediate their polarization towards M1 macrophages. Furthermore, DNA methyltransferase (DNMTs) is known to be involved in M2 differentiation and phenotypic regulation. Individuals with atherosclerosis and apolipoprotein E knockout mice fed an atherogenic diet displayed enhanced DNMT levels in macrophages (98). Since the activity of PPAR-γ is reduced, the macrophage transition from M1 to M2 is affected, and thus, the progression of atherosclerosis is marked by increased pro-inflammatory cytokine production. Oxidative stress is linked to increased histone acetyltransferase (HATs) activity of p300/CBP along with NFĸB DNA binding, promoting pro-inflammatory gene expression. In endothelial cells and hyperglycemic adipocytes, enhanced expression of HAT GCN5 and H3 acetylation is associated with increased ROS production, as confirmed in diabetic models. p300/CBP mediated acetylation of H3K9 at NOX2 promoter encourages ROS generation underlying complexity of epigenetic modifications in ROS balance and response (16).

Upon activation, neutrophils generate a range of ROS and O_2_^•-^ generated were reported to damage proteins and are limited to the compartment site it has been generated as its rate of dismutation was enhanced by SOD to H_2_O_2_. Neutrophil heme protein myeloperoxidase utilizes H_2_O_2_ to oxidize the halides chloride (Cl^-^), iodide (I^-^), and bromide (Br^-^), or pseudohalide anion thiocyanate (SCN^-^), into hypochlorous acid (HOCl) (99). Being a potent oxidant, HOCl poses a high reactivity towards biological macromolecules targeting free and protein-associated cysteine, methionine residues and low molecular weight thiols, and its ability to diffuse from the site of generation is very short. Thus, HOCl reacts with amines and forms chloramines, which are less reactive than HOCl and diffuse further from the generation site. The reaction of HOCl and chloramines with cytosine produces 5-chlorocytosine (5-clC), directly incorporated into DNA as a chlorinated nucleotide (100). Studies carried out reported gene silencing, and no significant changes were observed in global methylation levels due to the incorporation of 5-clC. Chloramines are also reported to interact with histone amine groups, thereby preventing methylation or acetylation events (101). However, the lack of *in vivo* experimental evidence makes it unclear how 5-clC and chloramine levels are regulated under physiological conditions. Neutrophil oxidants can react with cellular targets, including small molecules and redox-sensitive components of epigenetic pathways. Intracellular availability of methionine and ascorbate was depleted by neutrophil oxidants and reported to impact methylation by disrupting S-adenosylmethionine (SAM) levels (102). Mass spectrometry analysis to investigate the oxidant effects of methylation reported impaired cytosine methylation on newly replicated DNA in the Jurkat T-lymphoma cell line upon sub-lethal level exposure to glycine chloramines (103). The study reported DNMT1 inhibition and depletion of SAM levels at doses, which had minimal effect on cell proliferation. Though H_2_O_2_ treatment inhibited DNMT1, it did not reduce SAM or global methylation levels (104). Further experimentation is required to determine whether the methylation and demethylation effects observed are heritable to subsequent generations.

## 4 Summary and conclusion

A sustained pro-oxidant cellular environment mediates the development and progression of various pathological conditions due to redox imbalance. Dysregulation in these redox environments decreases the activity of mitochondria, TCA cycle and immune cell metabolism (30). ROS and RNS are ubiquitous byproducts of cellular metabolism, and any disparity between their generation and degradation in aging and diseases results in oxidative and nitrosative stress. Oxidative stress can irreversibly damage cellular structures, including membrane lipids or lipoproteins, forming oxidation-specific epitopes (OSE) on damaged cells (105). This damage-associated molecular pattern is recognized and removed by innate immune cells, including macrophages and neutrophils, enabling cellular homeostasis. Excessive accumulation of these oxidation products triggers chronic inflammation and metabolic disorders, including atherosclerosis, diabetes and age-related macular degeneration (106). Immune cells function and survival are regulated by various redox factors, including the intracellular and extracellular concentration of ROS/RNS and cellular antioxidants, namely glutathione, thioredoxin and Nrf-2 (107). Considering diabetes, metabolic imbalance in these conditions is characterized by increased glycolytic flux, and ROS act as a secondary messenger and mediates metabolic shift towards pro-inflammatory macrophage phenotype. ROS were also reported to activate multiple pro-inflammatory signaling pathways, including MAPK, NLRP3 and NFκB, resulting in an epigenetic modification in hyperglycemic conditions (108). A crosstalk between these immune cells and endothelial cells in diseased conditions is reported to stimulate increased ROS formation and inflammatory phenotypes further. Thus, consideration should be paved towards ROS generated by macrophages and neutrophils to suppress inflammation in metabolic disorders.

The functioning of individual immune cells is under redox control and reported to be sensitive to intracellular and extracellular concentrations of ROS and influenced by the activity of cellular antioxidants. Redox mechanisms regulate and modulate various immune functions, including metabolic reprogramming of dendritic cells (DCs), T cells, B cells, and natural killer cells (NK), aiding in its activation and regulation (109). ROS are reported to be involved in diverse biological events, including Epithelial–mesenchymal transition (EMT). This transdifferentiation process is vital in invasion and metastasis phenomena during neoplastic progression. ROS regulate the integrin arrangement and urokinase plasminogen activator (uPA) pathway in extracellular matrix remodelling (110). ROS can influence the function of various proteins involved in the EMT process through reversible or irreversible oxidative modification of protein on free cysteine residues (111). Thus, targeting redox regulation to prevent EMT and tumor metastasis is promising.

Mechanistic insight into the specific immune response generated for oxidation-specific epitopes at functional levels should be studied, which can aid in understanding oxidative stress and its associated chronic inflammations. Though M1 and M2 represent the two extreme phenotype characteristics of macrophage activation stages regulated by redox metabolism, tissue-resident macrophages comprise a distinct subset and hold tissue-specific functions dependent on oxygen and nutrient supply, which can certainly influence redox status and metabolism (112). Thus, studies focusing on the characterization of redox proteome during an immune response are essential. Methodology focusing on *in vivo* localization and visualization of ROS and their sources will aid in a better understanding of this complex redox metabolism in health and diseases.

## Author contributions

RRM: Conceptualization, Writing – original draft, Writing – review & editing. AP: Conceptualization, Project administration, Supervision, Writing – original draft, Writing – review & editing. PP: Writing – review & editing. JK: Conceptualization, Writing – original draft, Writing – review & editing.
